# Partial Correlation between Spatial and Temporal Regularities of Human Mobility

**DOI:** 10.1038/s41598-017-06508-1

**Published:** 2017-07-24

**Authors:** Wei Geng, Guang Yang

**Affiliations:** 1 School of Economics and Management, Southwest Jiaotong University, Chengdu, 610031 China; 2 Service Science and Innovation Key Laboratory of Sichuan Province, Chengdu, 610031 China; 3 Sichuan Airlines Co., Ltd., Chengdu, 610202 China

## Abstract

The regularity of human mobility has been extensively studied because of its prominent applications in a considerable number of important areas. Entropy, in addition to many other measures, has long been used to quantify the regularity of human mobility. We adopt the commonly used spatial entropy and develop an analogical temporal entropy to separately investigate the spatial and temporal regularities of human mobility. The underlying data are from an automated transit fare collection system operated by a metropolitan public transit authority in China. The distributions of both spatial and temporal entropies and their dependences on several widely used statistics are examined. The spatial and temporal entropies present a statistically significant correlation, which has not previously been reported to the best of our knowledge.

## Introduction

Intensive human displacement is driven by the fundamental needs of modern society, but such displacement consequently also reshapes society. Understanding human mobility is of great importance and has prominent applications in many areas, including, but not limited to, location-based services^1^, transportation^2–4^, migration^5^, epidemiology^6, 7^, disaster recovery^8, 9^, and even entrepreneurship^10^. Various data sources have been employed to improve the knowledge on human mobility. Mobile phone data, ranging from calling to texting and either individual or aggregated, have been extensively investigated in the literature due to their tractability on approximating human moving trajectories^11–17^. GPS trajectories^18^, location-based check-in data^19^, survey and interview results^10, 20, 21^, bank note circulations^22^, and census data^5, 14, 23^ also contribute to this body of literature. Another rich data source comes from automated transit fare collection systems, where smart cards are used to generate passengers’ exact spatiotemporal trajectories while completing fare transactions^2–4, 24–26^.

Regularity, also known as predictability, is the kernel of the current research on human mobility^13, 15^. Spatial regularity, which is closely related to the act of human displacement, has received a considerable amount of attention^6, 13, 15, 21, 22^. However, in many studies, the time dimension is considered as either a timescale in which human mobility is observed and measured^22^ or as a time order whereby human displacements are sequentially arranged for further investigation^15^. An emerging trend in the literature is focusing on temporal reoccurrence, i.e., temporal regularity, which harbours substantial potential. The latest advancements in this trend include explaining”familiar strangers” through the temporal regularity of daily bus transit^4^ and measuring the variability of temporal regularity as a function of time resolution^26^, among others.

Diverse types of measures, such as the probability distribution of displacements of a certain length^21, 22^, the probability distribution of the radius of gyration^13^ and the intensity of transit flows^6, 12^, have been developed to convey research on the regularity of human mobility, particularly along the spatial or spatiotemporal dimension. Moreover, the stream focusing on temporal regularity has some other measures, e.g., time variation of an individual’s daily transit known as absolute time difference^4^, inter-temporal difference of aggregated population between periods^16^, and accumulated variance based on the correlation between vectors at the aggregated level^26^. In addition to these measures, a more widely used quantity is entropy, which is analogous to the entropy that is well established in physics and information science. Entropy has long been used in spatial analysis and research^27–29^, and it is used to quantify the predictability of human spatial or spatiotemporal mobility^8, 15^.

In this paper, we separately investigate the spatial and temporal regularities of human mobility by calculating the spatial and temporal entropies for each individual. The underlying data are from an automated transit fare collection system operated by a transit authority in China. Compared to the data sizes reported in the literature, which range from hundreds^10, 18, 21^ to hundreds of thousands^19^ of individuals, our data set is fairly large, and it includes 5,759,234 trips of 195,065 individuals. Rich information from the data allows us to calculate spatial entropy as in the literature^8, 15^, as well as to calculate temporal entropies with appropriate time resolution, which is found to account for the variability of temporal regularity^26^. We examine the distributions of both spatial and temporal entropies, evaluate their dependences on several related factors, and test the correlation between spatial and temporal entropies. The obtained statistically significant correlation has not previously been reported to the best of our knowledge.

## Results

### Spatial and temporal regularities of individual mobility

We conduct our analysis based on passengers’ actual trips tracked by smart card transaction records. The data set contains 195,065 passengers’ daily transits in two months within a bus rapid transit system in Chengdu, China. For details about the data set, please refer to Methods.

**Figure 1:**
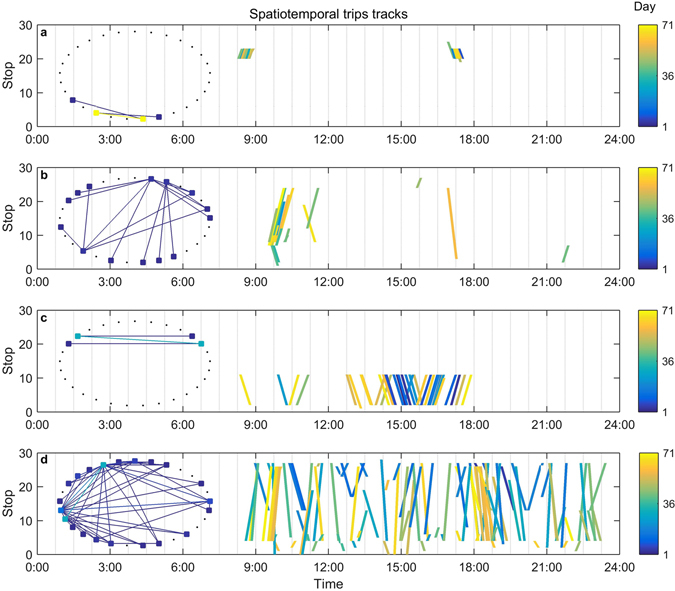
Individual spatiotemporal trip tracks. Each line segment in a specific panel represents a trip of the individual, where the left end indicates the trip origin and the right end indicates the trip destination. Every end point is located corresponding to its boarding or alighting time on the horizontal axis and stop on the vertical axis. The line segments are colour coded to illustrate the passage of time from day 1 to day 71 within the observation period. (**a**) The individual travels at an almost fixed time from a certain stop to another certain stop in the mornings and turns backwards in the afternoons. (**b**) The individual’s trips mostly occur at a stationary time but with diverse origins and destinations. (**c**) The individual always travels between two fixed stops but with a fairly large time diversity. (**d**) The individual’s trips are highly random. A subplot is provided in each panel to serve as a sketch map showing the origin-destination pairs of each individual with the colour bar omitted.

Remarkable heterogeneity in individual mobility could be observed from the data set. In Fig. 1, we present spatiotemporal trip tracks from four typical passengers with different travel patterns, which are distinguished from each other in terms of the dispersion of trip displacement and time interval. These figures reveal tremendous changes in spatiotemporal regularity across the entire population, and they also imply that a correlation between a single passenger’s spatial reappearance and temporal reoccurrence can hardly be achieved.

**Figure 2:**
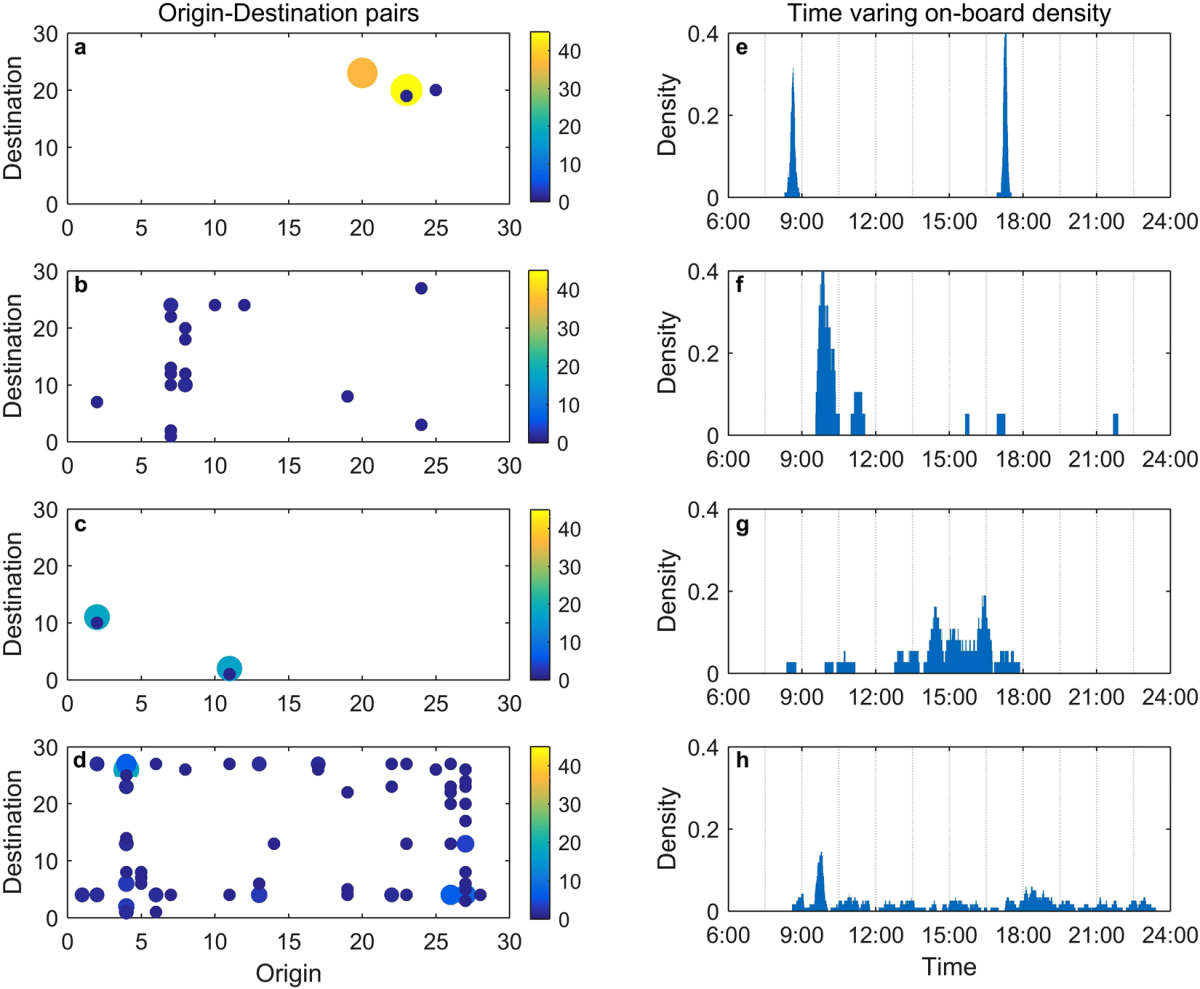
Individual spatial and temporal regularities. Each row corresponds to the individual in the same row in Fig. 1. (**a–d**) Each scattered dot represents an observed origin-destination pair with its area and colour showing the count of observations. Dots with larger areas and warmer colours represent a higher degree of spatial displacement regularity. (**e**–**h**) The empirical on-board density at each time is calculated by the on-board count at that time over the total number of observations. Narrower support with fewer sharp peaks suggests a higher degree of temporal recurrence regularity.

Having an anatomy of trips along either the spatial or temporal dimension provides further information. Panels (a)-(d) in Fig. 2 show origin-destination pairs with respect to repeating counts for the four individuals, and panels (e)-(h) show each individual’s empirical on-board time density. The eight panels serve as visualizations of those individuals’ spatial and temporal mobilities, which apparently have diverse patterns. The individual in the first row, i.e., panels (a) and (e), transits with high regularity in both the spatial and temporal dimensions. The individual in the second row transits with low spatial but high temporal regularity. The third individual transits with high spatial but low temporal regularity. The final individual transits with low regularity in both the spatial and temporal dimensions. These panels suggest that there is a high degree of heterogeneity in human mobility. To better characterize the heterogeneity and to conduct further analyses, measures of spatial and temporal mobilities should be introduced.

Inspired by Song *et al*.^15^, we follow the trend of using entropies to investigate the regularity of human mobility.

### Spatial entropy

The regularity of the passengers’ spatial displacement patterns could be well characterized by the temporal-uncorrelated entropy^15^, whose distribution serves as an indicator of heterogeneity in human spatial mobility. We relabel the temporal-uncorrelated entropy as spatial entropy in the remainder of this paper to highlight its role in measuring spatial mobility. In the context of displacement in a public transit system, two spatial locations are of great importance: the origin and the destination. Hence, we use both the origin and destination stops of each trip in formulating the spatial entropy. The definition of spatial entropy is provided in Methods.

**Figure 3:**
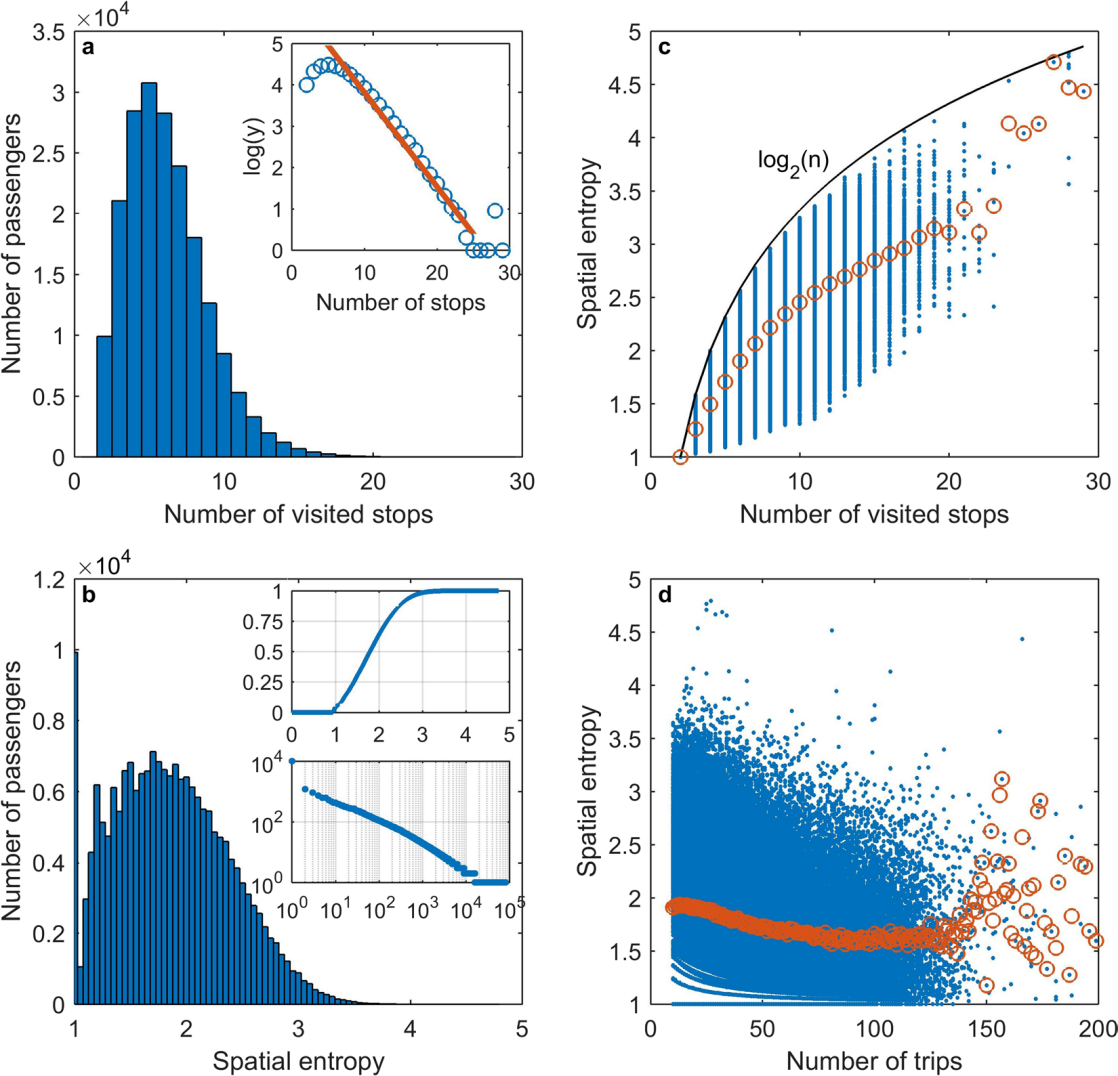
Spatial entropy and its distribution. (**a**) Each bar illustrates the number of passengers who visited a specific number of stops. As shown in the subplot, the right tail of the figure exponentially decreases. (**b**). The histogram shows the distribution of spatial entropy. The cumulative frequency distribution is presented in the upper subplot. The log-log graph in the lower subplot shows spatial transit patterns in descending order according to their counts in the entire population. (**c**) Dependence of spatial entropy on the number of visited stops. Each dot represents an individual. The theoretical upper bound log_2_(*n*) and the average entropies regarding each specific number of visited stops are also illustrated. (**d**) Dependence of spatial entropy on the number of trips. Each dot represents an individual. Each circle represents the average spatial entropy of passengers sharing a specific number of trips. For conciseness, a few outliers are not shown in the panels.

The spatial entropy is a function of the number of stops visited and the corresponding frequencies, both of which have diverse patterns at the individual level, as shown in Fig. 2. At the aggregated level, the number of stops visited follows an asymmetric unimodal distribution, as shown in Fig. 3(a), where its right tail exponentially decreases (log*y* = −0.2277*n* + 6.0895, p-value *p = *0, and coefficient of determination *R* ^2^ = 0.9801, excluding the last four observations). However, the spatial entropy, as shown in Fig. 3(b) in a histogram with a bin width of 0.05, has a multimodal distribution that appears as an asymmetric unimodal distribution with many fibres. The most significant fibre or peak appears at 1. A total of 5.09% of the passengers out of the entire population, who form the largest group with equal entropies at a single value, have their spatial entropies exactly equal to 1, which indicates that they travelled with high regularity between two specific stops with no exceptions. The upper subplot in Fig. 3(b) presents the cumulative distribution of the spatial entropies over the entire population. The median of the passengers’ spatial entropies is 1.8131. Passengers whose spatial entropy is 1.8131 transited in diverse patterns: half of them transited among 5 stops with probability [0.4667, 0.2667, 0.2000, 0.0333, 0.0333], 23.53% of them transited among 6 stops with probability [0.4545, 0.3636, 0.0909, 0.0455, 0.0227, 0.0227], and the others each transited in a different pattern. Details of those passengers’ transit patterns are presented in the Supporting Information. A total of 81,058 different transit patterns are present. These patterns are sorted in descending order according to the number of passengers who transited in the pattern and are shown in the lower subplot in Fig. 3(b) in a log-log graph, where the x-axis is the order and the y-axis is the number of passengers. Intuitively from the definition, spatial entropy may increase in the number of visited stops. We evaluate the situation and illustrate the dependence of spatial entropy on the number of visited stops in Fig. 3(c). The spatial entropy varies within a considerable range at each value of the number of visited stops *n*, although the average entropies regarding each specific number of visited stops exhibit an increasing trend. A comparison of the spatial entropies between individuals based on their values of *n* is therefore not performed in most cases. A tight upper bound log_2_(*n*) could be provided theoretically (see Methods), which is empirically a real tight bound for many values of *n* based on our data, indicating that some passengers actually travel among several different locations with equal frequencies. Figure 3(d) displays the dependence of spatial entropy on the number of trips, which is a statistic commonly used to label passengers as heavy or light users and to further distinguish their behaviours^30^. For the case of mobility regularity, the commonly used statistic is probably intuitively believed to have little impact since it only considers transit intensity. Those who should strictly transit between their homes and workplaces do not change their behaviours irrespective of how many trips they have accumulated. As shown in the figure, however, the spatial entropy gradually decreases in general as the number of trips increases. For clarity, we present the average entropy of passengers who share a specific number of trips to illustrate collective behaviour.

### Temporal entropy

A reasonable parallel concept on temporal regularity is temporal entropy, also known as spatial-uncorrelated entropy following the terminology used by Song *et al*.^15^. Given time slots and the probabilities of revisiting each time slot, the definition of temporal entropy is an exact analogue of that of spatial entropy, in which boarding or alighting time slots are used rather than stops. Details can be found in Methods. Since time moves continuously and only forward, we should select an appropriate aggregation level to form time slots, which is 30 minutes here, and consider recurrence on a quotient set, i.e., to identify time slots with the same temporal ends but on different days as the same one, which is also adopted in the literature^26^. Since the BRT system operates 17 hours per day, the grid size of 30 minutes yields a total of 34 time slots, which is comparable to the number of total stops (29) in the previous section.

**Figure 4:**
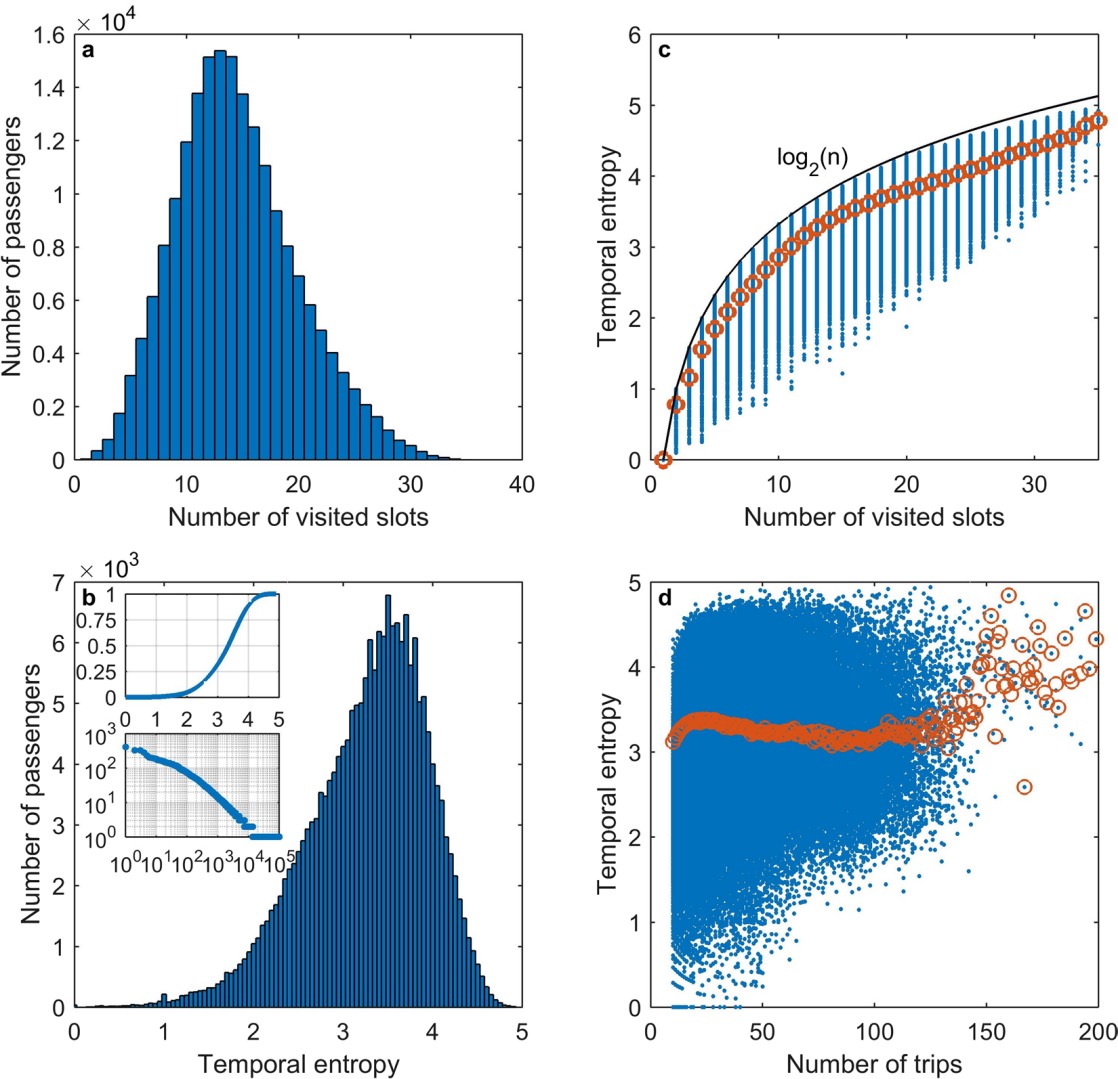
Temporal entropy and its distribution. (**a**) Each bar illustrates the number of passengers who visited a specific number of time slots with a grid size of 30 minutes. (**b**) The histogram of bin width 0.05 illustrates the distribution of temporal entropy. The cumulative frequency distribution is presented in the upper subplot. The log-log graph in the lower subplot shows temporal transit patterns in descending order according to their counts in the entire population. (**c**) The dependence of temporal entropy on the number of visited time slots is illustrated with a theoretical upper bound of log_2_(*n*). Each dot represents an individual. The circles represent the average entropies regarding each specific number of visited stops. (**d**) The dependence of temporal entropy on the number of trips. Each dot represents an individual. Each circle represents the average temporal entropy of individuals sharing a specific number of trips. For conciseness, a few outliers are not presented in the panels.

As in the previous section, we first evaluate the dependence of temporal entropy on the number of time slots visited, as shown in Fig. 4(a). The distribution is asymmetric with a heavier right tail. The distribution of temporal entropy, as shown in Fig. 4(b), has multiple modes. In contrast to the distribution of spatial entropy in Fig. 3(b), no remarkable peak appears to show any uniform transit pattern. The cumulative distribution of the temporal entropy is shown in the upper subplot. The median of the passengers’ temporal entropies is 3.3643. Passengers whose temporal entropy is 3.3643 transited in diverse patterns: nearly half of them transited among 13 time slots with probability [0.2500, 0.1429, 0.1071, 0.0714(×4), 0.0357(×6)], where (×*n*) denotes the number of repeating times; 11.76% of them transited among 12 time slots with probability [0.2500, 0.1071, 0.0714(×8), 0.0357(×2)]; 11.76% of them transited among 13 time slots with probability [0.2250, 0.1500, 0.1000(×2), 0.0750(×3), 0.0500(×2), 0.0250(×4)]; and the others each transited in a different pattern. Details of those passengers’ temporal transit patterns are presented in the Supporting Information. There are a total of 103,233 different temporal transit patterns. In the lower subplot, those patterns are sorted in descending order according to the number of passengers who transited in the pattern and are shown in a log-log graph, where the x-axis is the order and the y-axis is the number of passengers. We illustrate the dependence of temporal entropy on the number of visited time slots in Fig. 4(c). The temporal entropy varies within a considerable range at each value of the number of visited time slots *n*. Similar to the case of spatial entropy, a comparison of the temporal entropies between individuals sharing the same *n* is not performed in most cases. The tight upper bound log_2_(*n*) and average temporal entropies regarding each specific number of visited stops are also shown. The dependence of temporal entropy on the number of trips is shown in Fig. 4(d). As the number of trips increases, the average entropy of passengers who share the same number of trips first increases, then has a decreasing trend in general, and finally loses its trend when the number of trips increases so much that the sample size becomes too small.

### Spatial-temporal correlation at aggregated level

As we have already shown via some typical samples in Fig. 2, the spatial and temporal regularities may have diverse patterns and exhibit little correlation at the individual level. At the aggregated level, however, a different phenomenon emerges, similar to other behaviours reported in many previous works^21, 26^.

**Figure 5:**
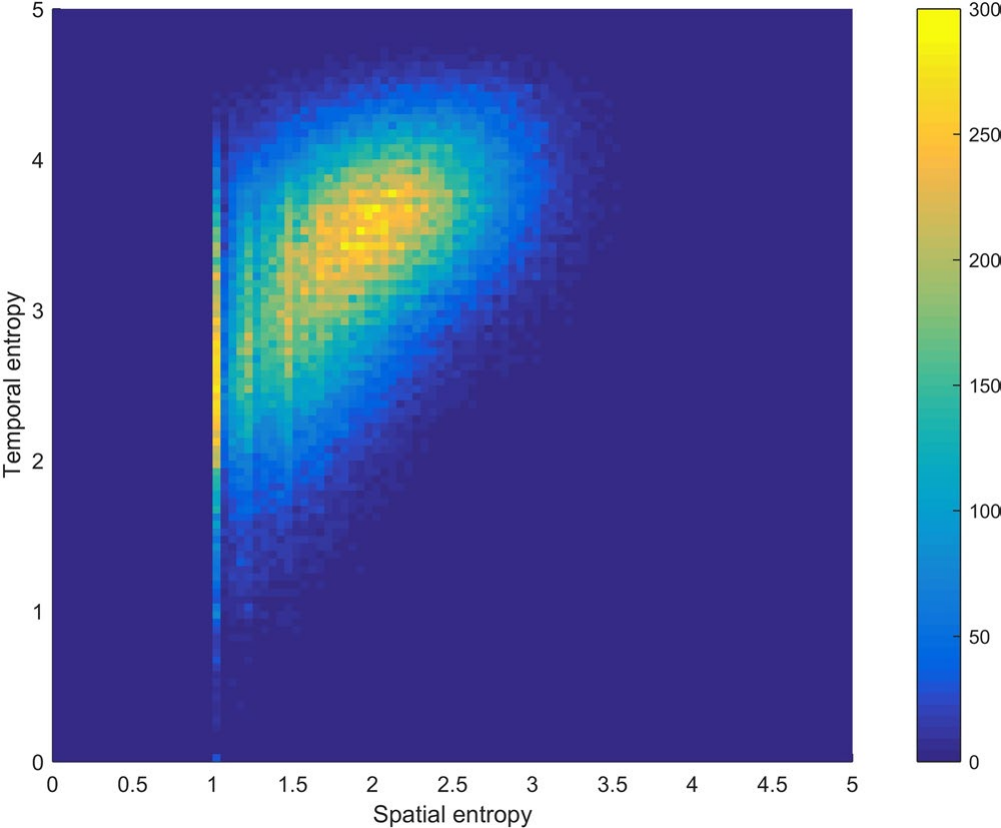
Spatiotemporal joint distribution. The heat map is produced based on a scatter plot, where every dot represents a passenger’s spatial-temporal regularity with its two coordinates being his or her spatial and temporal entropies, and multiple dots may overlap. The grid to support the heat map is 100 × 100 on [0, 5] × [0, 5], i.e., the grid size is 0.05. The degree of overlapping is visible according to different colours with the colour legend to the right of the heat map.

We locate individuals based on their spatial and temporal entropies to produce a heat map in Fig. 5. Passengers locating on the bottom left have lower values of both spatial and temporal entropies, implying that their transit behaviours have higher regularities, whereas passengers locating on the top right have larger values of entropies and behave less regularly. The entire population disperses more or less in a flipped drop shape, which has a narrow tail on the bottom left and gradually expands towards the top. The heat map highlights areas where most passengers’ spatial and temporal entropies locate. There are two distinct clusters observed in the figure. The first cluster is a very sharp band whose spatial entropies are 1 or very close to 1. The second cluster is an oblique band that leans from the lower left to the upper right around the point (2, 3.5). The oblique band suggests a correlation between the spatial and temporal entropies within this cluster. Regarding the spatial-temporal entropy pairs whose spatial entropies are not less than 1.05, which include 184,861 passengers (or 94.77% of the passengers), we conduct further statistical analyses. The ordinary least squares method provides *S* ^*t*^ = 0.6187*S* ^*s*^ + 2.1350 with p-value *p* = 0 and coefficient of determination *R* ^2^ = 0.2236. Meanwhile, the corresponding Spearman’s rank correlation coefficient between the two entropies is *ρ* = 0.4820 with p-value *p* = 0.

## Discussion

One of the major findings from the presented results is the correlation between spatial and temporal entropies over the vast majority of the entire population. The alternative name for our spatial entropy in the literature^15^, i.e., temporal-uncorrelated entropy, indicates that its definition includes no explicit term concerning any temporal mobility. The temporal entropy defined in this paper, however, includes no explicit term concerning any spatial mobility. Consequently, the two entropies respectively measure human mobility along two uncorrelated dimensions. The significant correlation between the two so-defined entropies over the vast majority of the entire population probably suggests that scholars should be cautious when they are attempting to adopt spatial entropy to control the temporal effect on human mobility, or vice versa. The significant correlation between the two entropies also indicates that some degree of consistency between spatial and temporal human mobilities is present, which relates to the nature of human behaviour. Our finding contributes new evidence from the area of human mobility, where the correlation between spatial and temporal regularities has not previously been reported. This evident correlation may also shed light on future studies. The factors that determine the correlation between spatial and temporal human mobilities are not yet clear. Further studies based on carefully designed and performed field experiments should be conducted to eliminate the noise of possible spurious correlations and to identify the origin of the present correlation. Moreover, the correlation that we found and reported is at the collective level; however, it should have its root in individual decision making and behaviours. As extensively studied in psychology, consistency widely exists among human thoughts, feelings and actions^31^. Human mobility, irrespective of how we measure its regularity, is a type of human action or behaviour motivated by some inherent attitudes and personalities and should exhibit consistency to some degree. More research could be conducted to further advance our understanding of this topic.

Correlation is not the only story. Human mobility within a metropolitan public transit system is subject to urban planning, shift schedules, and many other outside influences. Therefore, a noticeable partial inconsistency between spatial and temporal entropies also exists. The dispersion of passenger locations on the map of spatial-temporal entropies in Fig. 5, which has a flipped drop shape, evidently also supports the partial inconsistency. Outside the major band conveying consistency, the top left area of this plot is evidence of partial inconsistency, in which passengers probably transit with high regularity among few stops but with considerably less regularity in many of the time slots.

Extensive studies^11, 13, 15, 21^ have found that a great majority of individual displacements occur among a few dominant locations, such as home, school and workplace, whereas a long tail exists where the individual visits many other locations with very small frequencies. Less efforts, however, have been focused on examining these individuals’ temporal regularities. Our findings reveal significant heterogeneity within the aforementioned group in terms of temporal entropy, which is worth investigating further. It could be inferred that spatial constraints are relatively tighter for individuals that are studying, working, or conducting some other affairs, whereas temporal constraints are more flexible to offer individuals a larger degree of freedom in daily transit. Further discussions on urban planning, labour force policy, and work design could be constructed thereafter.

## Methods

### Data description

Our data are from Chengdu, one of the top metropolitan areas in China, which has installed and operated a loop BRT system that connects several major residential and commercial areas since 2013. Two BRT lines, which respectively run clockwise and anti-clockwise, with 29 stops serve all the passengers in this area.

The data consist of all trips paid for using local smart cards, namely, Tianfutong, from Jun. 11, 2013, to Aug. 20, 2013. We conducted a data screening process prior to the analysis. The first step is to remove those users who travel less than 10 trips, which is similar to the process in the literature^4^ and is performed to avoid insignificance and error from small sample outliers. The second step is to remove those users who have at least one trip whose origin and destination are the same stop to focus on ordinary travel behaviours. After the data screening process, we observe that a total of 195,065 users travelled in the BRT system during the aforementioned time period and that the overall number of trips was 5,759,234. For each trip, the following information is collected: origin, destination, boarding time, alighting time, and direction (clockwise or anti-clockwise). Trip duration, or dwell time, could subsequently be calculated. Stops are labelled with numbers from 1 to 29 for identification.

Similar to many rail transit systems, the BRT authority in Chengdu requires passengers to onboard or alight the vehicle in a paid area at every stop. Consequently, the automatic fare collection system actually tracked the times when the passengers entered or exited the paid area.

Throughout this paper, we do not separate the data to analyse passengers’ transit behaviours on weekdays and weekends. Some analyses based on separated data are presented in the Supporting Information. Briefly, the entropies on all days significantly depend on the entropies on weekdays, and the findings based on data from weekdays are similar to those reported in the previous Results section.

### Entropy

For an arbitrary passenger *i*, assume that he or she gets on board or gets off at *N*_*i*_ stops in total and visits any stop *j* out of the *N*_*i*_ stops with an empirical probability *p*_*i*_(*j*). The spatial entropy of passenger *i* is then

1 $${S}_{i}^{s}=-\sum _{j=1}^{{N}_{i}}{p}_{i}(j){\mathrm{log}}_{2}{p}_{i}(j).$$

To well define temporal entropy, time slots and the probability of revisiting are two preliminary issues.

The time stamp in our data has a resolution of up to one second, which provides great convenience for adjusting and choosing an appropriate scale or grid size for the time slot. When calculating a spatiotemporal entropy, a grid size of one hour was chosen by Song *et al*.^15^, possibly to avoid a cumbersome state space. The grid size chosen by Sun *et al*.^16^ was 5 minutes, which is exactly the temporal resolution of their data. Meanwhile, Zhong *et al*.^26^ used grid sizes ranging from 1 minute to 12 hours to meet the needs of testing variability. For our purposes, we consider the grid size of 30 minutes.

Time only moves forward; hence, it is impossible to revisit any time slot of appropriate length. An alternative approach is to consider recurrence on a quotient set of time slots in one single day. In other words, we identify time slots with the same temporal ends but on different days as the same one, which is also adopted in the literature^26^.

For an arbitrary passenger *i*, assume that he or she gets on board or gets off at *M*_*i*_ time slots in total, and for any time slot *j* out of the *M*_*i*_ time slots, an empirical probability *p*_*i*_(*j*) applies. The temporal entropy of passenger *i* is then

2 $${S}_{i}^{t}=-\sum _{j=1}^{{M}_{i}}{p}_{i}(j){\mathrm{log}}_{2}{p}_{i}(j).$$

### Boundaries

For an arbitrary passenger *i*, assume that he or she gets on board or gets off at *n* stops in total. According to the definition of spatial entropy, it is easy to determine that ${S}_{i}^{s}$ obtains its maximum value if and only if the passenger visits the *n* stops with equal probability 1/*n*. Therefore,

3 $${S}_{i}^{s}=-\sum _{j=1}^{n}{p}_{i}(j){{\rm{l}}{\rm{o}}{\rm{g}}}_{2}{p}_{i}(j)\le -\sum _{j=1}^{n}\frac{1}{n}{{\rm{l}}{\rm{o}}{\rm{g}}}_{2}\frac{1}{n}={{\rm{l}}{\rm{o}}{\rm{g}}}_{2}(n).$$

Assume that *p*_*i*_(*j*) approaches 1 for an arbitrary *j*; it is easy to find that ${S}_{i}^{s}$ approaches 0, although it cannot reach 0 unless *n* = 1. Hence, for any *n* > 1, ${S}_{i}^{s}$ > 0.

Similarly, ${S}_{i}^{t}$ > 0 for any *n* > 1 and ${S}_{i}^{t}$ ≤ log_2_(*n*).

### Data availability

The data that support the findings of this study are available from Chengdu Public Transport Group Co., Ltd, but restrictions apply to the availability of these data, which were used under license for the current study and are thus not publicly available. However, the data are available from the authors upon reasonable request and with permission of Chengdu Public Transport Group Co., Ltd.

## Electronic supplementary material

Supplementary Information
