# Supplementary material for: Partial Correlation between Spatial and Temporal Regularities of Human Mobility
Source: Sci Rep. 2017 Jul 24;7:6249. doi: 10.1038/s41598-017-06508-1 (PMC5524686; doi:10.1038/s41598-017-06508-1)
Supplement: Supplementary file 1 — Supplementary Information [file 41598_2017_6508_MOESM1_ESM.pdf]

Supporting Information
Partial Correlation between Spatial and Temporal Regularities of Human Mobility

Wei Geng[1,2,*], Guang Yang[1,3]

1. School of Economics and Management, Southwest Jiaotong University, Chengdu 610031, China

2. Service Science and Innovation Key Laboratory of Sichuan Province, Chengdu 610031, China

3. Sichuan Airlines Co., Ltd., Chengdu, 610202, China

* Corresponding author: wgeng@swjtu.edu.cn

1 BRT system in Chengdu

The Bus Rapid Transit (BRT) system we observed locates in Chengdu, one of the top metropolitan areas in China. The system have been operated to connect several major residential and commercial areas since 2013. Running on the city's Second Ring Elevated Road, the BRT system has a circle route with 29 stops as shown in the sketched map in Fig. 1.

It's of great importance to understand origin-destination (OD) flows for public transit planning and operations. We visualize the OD matrix in the following Fig. 2. It is color-coded according to the flow on each OD pair. Panel (a) exhibits flows from stops with smaller numbers to those with larger ones, whereas panel (b) exhibits flows oppositely. No loop or ring trip is considered, hence there is no OD pair from an arbitrary stop to itself. It is readily seen that the two graphs are not identical, i.e., the OD matrix was not symmetric. Moreover, the graphs illustrate that traffic in the BRT system was far away from uniformly distributed among the stops. Certain stops, e.g., stop 20 and 29, attracted major traffic both entering and exiting the system. Stops located in southern and southwestern part of the system served the highest portion of demand compared to other stops. It provides hints for further planning and possible operational improvements such as operating express routes to bypass some less used stops, or providing short run routes to only serve those stops with high loads especially in rush hours.

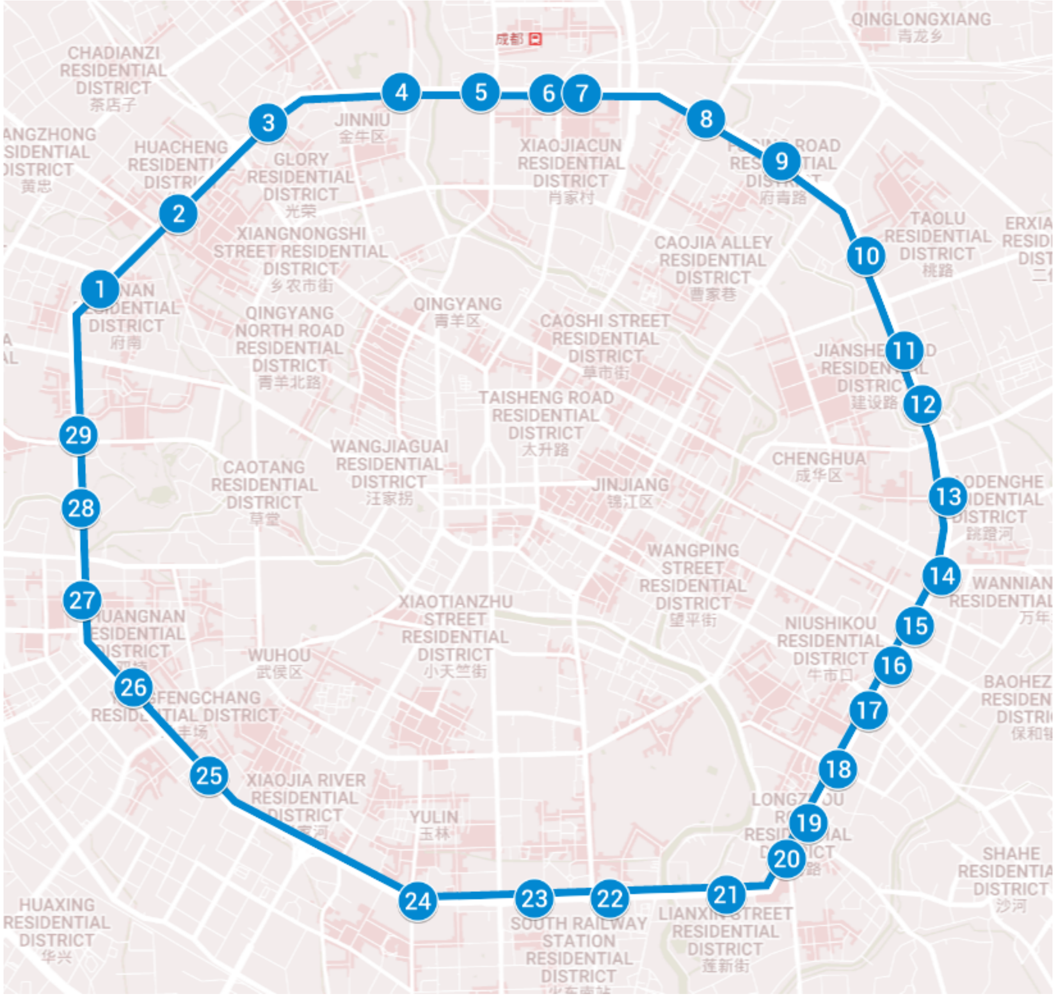

Figure 1: **BRT route and stops.** The BRT route is sketched in blue line. Each blue dot with a number represents a stop. There are 29 stops in total. The map data is provided by Google. The map with stop labels could be accessed on Google Maps.

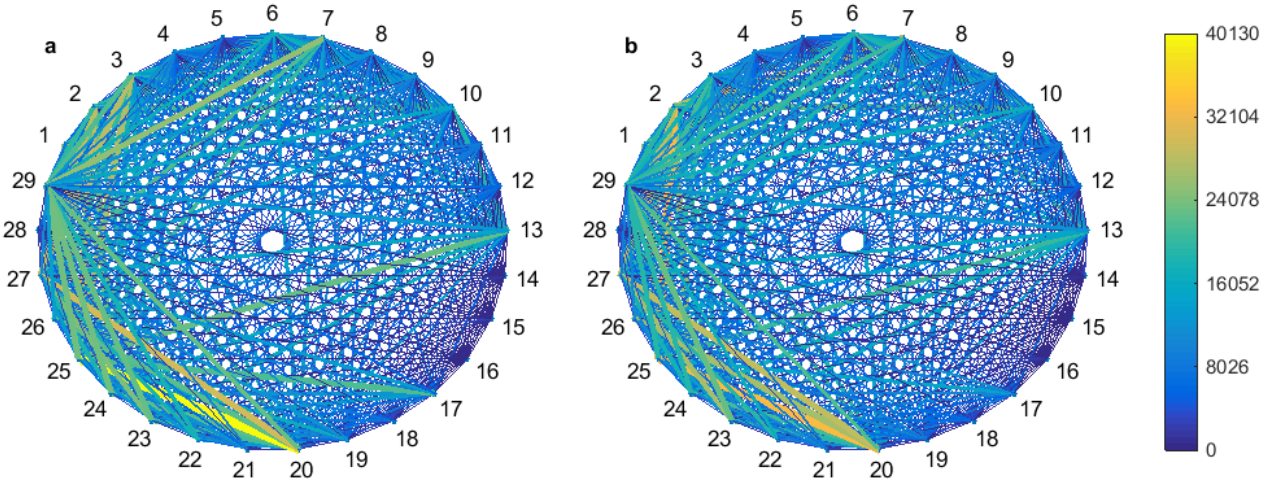

Figure 2: **Origin-Destination flows.** (a) Trips from smaller numbered stops to larger numbered ones. (b) Trips in opposite directions.

2 Passenger transit patterns

Entropy has been extensively used to measure regularities of human mobility. It provides a simple and easy-to-use quantitative metric for many applications. Recalling the definition of either spatial or temporal entropy in the section **Method** of the main body, any one of the two entropies is in fact a function mapping passenger transit patterns into nonnegative real numbers. Some detailed information may be dropped during the mapping process. And regarding a specific value of entropy, multiple passenger transit patterns may present. However, for the purpose of our research, spatial or temporal entropy is good to describe the regularities of human mobility due to their conciseness, whereas passenger transit patterns is pretty cumbersome.

Passenger transit patterns behave better, however, in describing human mobility in a clear and striking manner. In the main body of the manuscript, we reported some major passenger transit patterns of those whose spatial or temporal entropy equals the median value over the whole population. Here we provide detailed information in the following two tables. Table 1 exhibits spatial transit patterns, and Table 2 exhibits temporal transit patterns.

Table 1: Spatial transit patterns of passengers whose entropy equals the population median

Counts	Number of visited stops	Visiting probability
17	5	0.4667 0.2667 0.2000 0.0333 0.0333
8	6	0.4545 0.3636 0.0909 0.0455 0.0227 0.0227
1	5	0.3871 0.3065 0.2581 0.0323 0.0161
1	5	0.4348 0.3696 0.0870 0.0652 0.0435
1	6	0.4767 0.3023 0.1512 0.0349 0.0233 0.0116
1	6	0.4839 0.2742 0.1774 0.0323 0.0161 0.0161
1	6	0.4746 0.2966 0.1610 0.0339 0.0254 0.0085
1	7	0.4904 0.3173 0.0865 0.0769 0.0096 0.0096 0.0096
1	7	0.4891 0.2663 0.1848 0.0272 0.0163 0.0109 0.0054
1	8	0.4394 0.4091 0.0606 0.0303 0.0152 0.0152 0.0152 0.0152
1	12	0.4557 0.4177 0.0316 0.0190 0.0127 $(\times 4)^*$ 0.0063 $(\times 4)^*$

* $\times n$ indicates its repeating times.

3 Passenger behaviours in weekdays and weekends

Difference between passengers transit behaviours in weekdays and weekends has been extensively investigated in literature. Sun et.al. (2013) discussed this issue and provided collective evidence in their supporting information appendix. Others concerning this issue include Roth et.al. (2011); Liang et.al. (2012); Zhong et.al. (2016).

Table 2: Temporal transit patterns of passengers whose entropy equals the population median

Counts	Number of visited slots	Visiting probability
8	13	0.2500 0.1429 0.1071 0.0714(×4)* 0.0357(×6)*
2	12	0.2500 0.1071 0.0714(×8)* 0.0357(×2)*
2	13	0.2250 0.1500 0.1000(×2)* 0.0750(×3)* 0.0500(×2)* 0.0250(×4)*
1	13	0.1875 0.1375 0.1125 0.1000(×2)* 0.0875 0.0750(×2)* 0.0500 0.0375 0.0125(×3)*
1	13	0.1667(×2)* 0.1429 0.1190 0.0952 0.0714 0.0476(×3)* 0.0238(×4)*
1	14	0.2500 0.1429 0.1429 0.1071 0.0357(×10)*
1	17	0.2750 0.1500 0.1250 0.1000 0.0750 0.0500 0.0375 0.0250(×5)* 0.0125(×5)*
1	22	0.3133 0.1687 0.1084 0.0843 0.0663 0.0361 0.0301(×3)* 0.0120(×9)* 0.0060(×4)*

* $\times n$ indicates its repeating times.

In the main body of the manuscript, we focused on general spatial or temporal regularities of human individuals, and do not intend to distinguish difference in regularities between weekdays and weekends. Therefore, we did not separate our dataset to conduct analysis depending on weekdays and rest days. The approach is also common in literature, e.g., Yan et.al. (2013); Palchykov et.al. (2014); Zhao et.al. (2015). The separation, however, brings more information which may benefit our readers. Hence, we present detailed results on separated datasets here.

The following Table 3 shows some summary statistics of the entropies in all days and in weekdays. Each of the three statistics (population median, mean and standard deviation) of either spatial entropy or temporal entropy in all days and in weekdays are of comparable quantities. We next illustrate individual level data and calculate correlations between entropies in all days and in weekdays.

Table 3: Summary statistics

	Spatial entropy		Temporal entropy	
	All days	Weekdays	All days	Weekdays
Count	195,065	194,520	195,065	194,520
Median	1.8131	1.7037	3.3643	3.0660
Mean	1.8506	1.7483	3.2703	3.0008
Standard deviation	0.5194	0.5023	0.6722	0.7076

194,520 passengers ever travelled in weekdays. The following Fig. 3 illustrates the dependence of their entropies in all days on those in weekdays. As shown in the panel a, a remarkable number

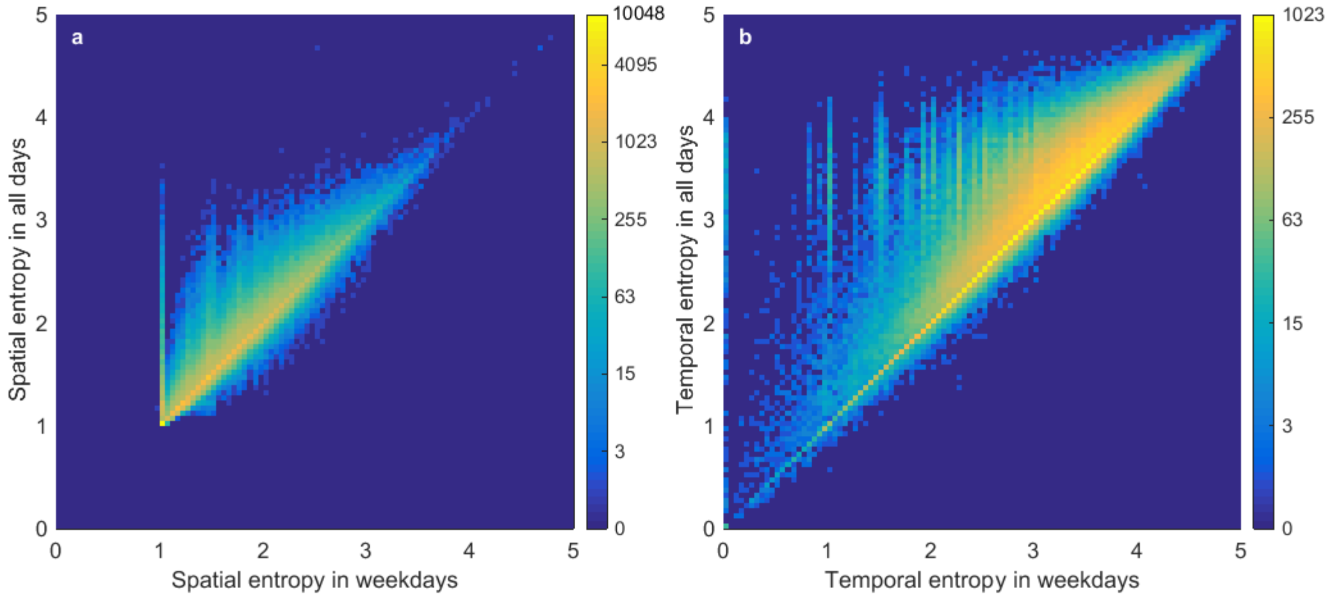

Figure 3: **Dependence of entropies in all days on those in weekdays.** The heatmaps are produced on a 100×100 grid on $[0, 5] \times [0, 5]$, i.e., the grid size is 0.05. (a) Dependence of spatial entropies in all days on those in weekdays. (b) Dependence of temporal entropies in all days on those in weekdays.

of passengers have their spatial entropies in all days (SA, for short) equal to those in weekdays (SW), and a notable linear trend presents between SA and SW. And panel b exhibits similar phenomenon regarding to temporal entropies in all days (TA) and in weekdays (TW). The OLS method reports that SA has a significantly linear dependence on SW (p-value $p = 0$, coefficient of determination $R^2 = 0.8540$), and TA has a significantly linear dependence on TW (p-value $p = 0$, coefficient of determination $R^2 = 0.7328$). Furthermore, the Spearman's rank correlation coefficient between SA and SW is $\rho = 0.9204$ with p-value $p = 0$, and that between TA and TW is $\rho = 0.8618$ with p-value $p = 0$.

Since either spatial entropies or temporal entropies in all days are significantly correlated with those in weekdays, the results we reported based on unseparated dataset in the main body of the manuscript are highly similar as those based on separated datset which only include passengers' transits in weekdays.

References

Liang, X., Zheng, X., Lv, W., Zhu, T. and Xu, K., 2012. The scaling of human mobility by taxis is exponential. *Physica A: Statistical Mechanics and its Applications*, 391(5), pp.2135-2144.

Palchykov, V., Mitrović, M., Jo, H.H., Saramäki, J. and Pan, R.K., 2014. Inferring human mobility using communication patterns. *Scientific Reports*, 4.

Roth, C., Kang, S.M., Batty, M. and Barthélemy, M., 2011. Structure of urban movements: polycentric activity and entangled hierarchical flows. *PloS one*, 6(1), p.e15923.

Sun, L., Axhausen, K.W., Lee, D.H. and Huang, X., 2013. Understanding metropolitan patterns of daily encounters. *Proceedings of the National Academy of Sciences*, 110(34), pp.13774-13779.

Yan, X.Y., Han, X.P., Wang, B.H. and Zhou, T., 2013. Diversity of individual mobility patterns and emergence of aggregated scaling laws. *Scientific Reports*, 3.

Zhao, K., Musolesi, M., Hui, P., Rao, W. and Tarkoma, S., 2015. Explaining the power-law distribution of human mobility through transportation modality decomposition. *Scientific reports*, 5.

Zhong, C., Batty, M., Manley, E., Wang, J., Wang, Z., Chen, F. and Schmitt, G., 2016. Variability in regularity: Mining temporal mobility patterns in london, singapore and beijing using smart-card data. *PloS one*, 11(2), p.e0149222.
